# Effectiveness of Telephone Interventions for the Management of Behavioral and Psychological Symptoms of Dementia in the Community: Systematic Review

**DOI:** 10.2196/77233

**Published:** 2025-10-20

**Authors:** Angela Cebolla Sousa, Geva Greenfield, Pallavi Nair, Reham Aldakhil, Judith Udoyeh, Manisha Karki, Aos Alaa, Eva Riboli-Sasco, Austen El-Osta, Ana Luisa Neves, Benedict Hayhoe

**Affiliations:** 1 Department of Primary Care and Public Health, School of Public Health, Imperial College London, 90 Wood Ln, London, W12 0BZ, United Kingdom, +44 20 7589 5111; 2 Self-Care Academic Research Unit, School of Public Health, Imperial College London, London, United Kingdom

**Keywords:** behavioral and psychological symptoms of dementia, caregiver burden, hospitalizations, telephone interventions, systematic review, BPSD

## Abstract

**Background:**

Most people living with dementia experience behavioral and psychological symptoms of dementia (BPSD), leading to poor quality of life and hospitalizations and causing a significant burden for informal caregivers and health care systems, with a global lack of equitable support to manage these symptoms in the community. Telephone interventions can potentially improve the accessibility and flexibility of long-term dementia support.

**Objective:**

This systematic review evaluates the effectiveness of telephone interventions in managing BPSD for community-dwelling patients with dementia and their informal caregivers, and thereby reducing BPSD-related hospitalizations.

**Methods:**

A systematic search of 4 databases (MEDLINE, Embase, PsycInfo, and SCOPUS) was conducted. The authors included studies with telephone interventions with no blended component (ie, other technologies or in-person portion) and outcomes assessing the impact of these interventions on people with dementia, informal caregivers, and hospitalizations using quantitative measures. The risk of bias of the studies was measured using the National Heart, Lung, and Blood Institute assessment tools. Findings were analyzed applying a thematic synthesis approach.

**Results:**

Of 4355 studies screened in 2024, 12 met the inclusion criteria. Studies were conducted in 5 high-income countries, and the majority were randomized controlled trials, with 2 non-randomized controlled trials and 2 pre-post intervention studies. Interventions included telephone coaching calls, psychosocial and educational support calls, and online platforms. Most studies showed a reduction in BPSD and BPSD-related burden; however, the certainty of this evidence was rated as low according to the GRADE (Grading of Recommendations Assessment, Development and Evaluation) analysis. In total, 9 studies reported reduced BPSD, and 5 studies showed a statistically significant decrease, while 4 studies indicated significant improvements in BPSD-related caregiver burden. One study considered BPSD-related hospital admissions, reporting a statistically significant reduction in admission rates.

**Conclusions:**

Telephone interventions delivered through psychosocial and educational calls and online platforms are promising tools for reducing BPSD-related caregiver burden. Personalized telephone interventions, including patients and informal caregivers in the treatment plan, may improve behavioral and psychological symptoms in patients with dementia. However, the certainty of evidence for both outcomes was low; therefore, these findings should be interpreted with caution. To strengthen the evidence base and assess the global applicability of such interventions, high-quality studies—particularly in low- and middle-income countries—are needed. Future research should incorporate longer follow-up periods, cost-effectiveness analyses, and greater consistency in intervention design and outcome measurement to better inform clinical practice and policy.

## Introduction

Dementia affects approximately 55 million people worldwide—a number projected to increase to 139 million by 2050 [1]. Dementia creates a significant economic, social, and health care burden globally, with a global cost of approximately US $1.3 trillion per year [2].

A major source of dementia-related health care burden is the behavioral and psychological symptoms of dementia (BPSD) [3], which are predominantly anxiety, depression, apathy, hallucinations, agitation, and aggression. It is estimated that 97% of people living at home with dementia will experience BPSD, with their severity increasing as the disease progresses [4]. BPSD has biopsychosocial root causes, including unmet needs and inadequate environmental conditions [4]. These neuropsychiatric symptoms have far-reaching consequences, as they profoundly impact both the individuals themselves and their support networks, amplifying the burden on carers, most of whom are unpaid family members or friends [4,5]. BPSD also acts as a catalyst for increased hospitalizations, costs of care, and premature institutionalizations [3-5].

Nonpharmacological interventions are considered the first-line treatment for BPSD and can often be administered at home [6]. However, studies have highlighted the need for enhanced community-based support for patients with dementia and their informal caregivers to ensure that they receive adequate education and long-term help to manage BPSD at home [6,7]. Despite this, at least two-thirds of informal caregivers, particularly in low- and middle-income countries (LMICs), encounter barriers in accessing sufficient community support services. This may be due to service costs, lack of time, stigma, place of residence, or systemic issues, such as inadequate signposting to these services [8,9].

Digital technologies can potentially reduce this gap and improve the accessibility, flexibility, and continuity of support for managing BPSD in the community. For instance, these technologies can help families living in remote areas or those with time or mobility constraints to access long-term dementia care support and improve BPSD monitoring [10].

Previous systematic reviews have shown promising results from the use of digital technology to manage BPSD [11-13]. These tools had the potential to improve BPSD, help caregivers address these symptoms at home, and alleviate their burden. However, the digital technologies used in these studies can often be inaccessible for specific population groups and in different resource settings across countries due to differences in health care and technological resources or staff, among other reasons [14]. In addition, some patients and caregivers may lack the digital literacy to use these technologies effectively, limiting equity in access and reducing their scalability [14,15].

No systematic review has, however, summarized the impact of telephone interventions, the most widely used technology, in the management of BPSD in the community. Nearly 75% of the global population has access to a telephone, including almost 50% in low-income countries [16]. Telephones are well used and accepted by the older population, especially compared to more complex technologies [17]. This pervasive communications modality can offer a lower-cost and more accessible solution to manage complex diseases, such as dementia worldwide. Telephones can also provide the opportunity for people living in remote areas or for those encountering other environmental barriers to health care to access more continuous dementia care support [18,19]. Furthermore, an essential part of dementia care was necessarily provided through telephones across countries during the COVID-19 pandemic and ensuing national lockdowns [19,20]. Examining the effects of telephone interventions could also help accelerate the accomplishment of the World Health Organization’s (WHO) "Global Action Plan on the Public Health Response to Dementia" of providing equitable dementia care internationally [21]. Likewise, in countries such as the United Kingdom, these interventions align with the UK National Health Service (NHS) long-term plan, which aims to increase the implementation of digital tools for lifelong care [22]. A summary of the effectiveness of telephone interventions in the management of BPSD in the community can help fill an important literature gap and provide evidence to address this major global health issue.

This systematic review aims to summarize the impact of telephone interventions designed for community-dwelling adults living with dementia and their informal caregivers on (1) the frequency, severity, and intensity of BPSD; (2) BPSD-related caregiver burden; and in (3) BPSD-related hospital admissions. It is anticipated that this could help guide future practice, research, and policy on the usage of telephones to improve the management of BPSD in the community.

## Methods

### Protocol Development and Registration

A systematic review was conducted according to the PRISMA (Preferred Reporting Items for Systematic reviews and Meta-Analyses) guidelines in Checklist 1 [23] and registered in the PROSPERO (International Prospective Register of Systematic Reviews; protocol number CRD42024521363). A member of the public who was a carer of a person with dementia was involved during the conceptualization of the study protocol. Amendments made to the original protocol can be found in the revision notes of the protocol in the PROSPERO.

### Literature Search

A comprehensive search with the assistance of an experienced medical librarian was conducted using 4 databases, such as MEDLINE (Ovid), Embase (Ovid), PsycInfo (Ovid), and SCOPUS. Both keywords and medical subject headings were included to ensure that the search was exhaustive.

We based the main categories of the search strategy on the PICOS (Population, Intervention, Comparison, Outcomes, and Study Design) framework, using the following main terms: (1) dementia, (2) BPSD, and (3) telephone interventions. The former included all types of dementia, and BPSD comprised terms specific to the individual symptoms. The latter incorporated mobile health apps, electronic consultation, or telehealth delivered through a telephone. The search strategy was inspired by 2 other previous systematic reviews with a similar methodology [12,24]. The full search strategy for each database can be found in the Multimedia Appendix 1. The studies’ extraction from the databases was performed on March 14, 2024. There was no limit on the date range applied to the publications extracted. No gray literature was sought.

### Study Selection

Five reviewers (AA, ACS, ERS, JU, and MK) performed the title and abstract screening of each of the 4355 papers (2 reviewers per paper) in Covidence (Veritas Health Innovation Ltd) independently. Conflicts between reviewers were resolved through discussion with a further researcher (PN). Three independent reviewers (ACS, JU, and RA) performed the full-text review of each study (2 reviewers per study), and the conflicts were discussed between these reviewers. Studies that did not have a full-text version available were excluded. Those studies that did not explicitly mention the type of technology used were also removed to reduce the uncertainty of the results for this review. Neither time nor language restrictions were applied. When papers were written in a language other than English, the authors searched for an available English version. Table 1 shows the criteria used and the rationale behind each of them. Multimedia Appendix 2 has further details on the reasoning behind each selection criterion.

**Table 1.** Inclusion and exclusion criteria for the systematic review.

Component	Inclusion	Exclusion	Rationale
Population	Community-dwelling people with dementia Their informal caregivers	Patients with dementia living in care or nursing homes Formal caregivers	These specific population groups were chosen for the study, as most patients with dementia live at home worldwide. In the same way, most caregivers are informal globally [5].
Intervention	Telephone-based interventions designed for people with dementia and their informal caregivers. These would range from mobile health apps to telemedicine delivered through a phone.	Blended interventions, that is, having an in-person component or combined with other technologies.	They are the most highly accessible digital technology worldwide [16]. In the same way, these interventions do not always require a broadband connection to work, unlike other digital technologies.
Control	N/A^a^	N/A	N/A^b^
Primary Outcomes	Change in BPSD^c^ in community-dwelling adults Change in BPSD-related informal caregiver burden Change in BPSD-related hospitalizations	Change in other related outcomes, such as social isolation or quality of life of informal caregivers or adults with dementia. Difference in caregiver burden or hospitalizations not related to BPSD.	BPSD is largely associated with a decrease in quality of life both in patients living with dementia and their informal carers, poorer health outcomes, as well as more frequent hospitalizations. The first-line treatment recommended for BPSD is nonpharmacological interventions [6].
Study design	All forms of design, including quantitative measures. Mixed methods studies, as long as the findings could be extracted from the quantitative strand.	Qualitative Case studies and series Intervention modeling studies	Qualitative studies were excluded due to the study aims. Case studies and series were also excluded because of their lower reliability.

a N/A: Not applicable.

b Observational studies were not excluded from the selection criteria of studies.

c BPSD: behavioral and psychological symptoms of dementia.

### Risk of Bias Assessment

Risk of bias was assessed using the study design–specific tools available from the National Heart, Lung, and Blood Institute (controlled trials or pre-post study intervention scales). This tool provides a set of questions to evaluate the internal validity of studies in systematic reviews, including randomization of participants, dropout rates, and outcome measurement. Studies were classified as poor, fair, or good, depending on the number of criteria they fulfilled according to the authors’ judgments and their study design [25]. Studies were not excluded based on the outcome of the risk of bias assessment. ACS conducted these assessments, which were reviewed independently by 5 coauthors (ALN, AEO, BH, GG, and PN). This risk of bias assessment was subsequently incorporated into the GRADE (Grading of Recommendations Assessment, Development and Evaluation) certainty of evidence evaluation.

### Data Extraction and Analysis

The data were extracted by ACS. Results were collected using a data extraction table in Excel (Microsoft Corporation). The final results were reviewed by the other authors independently (ALN, AEO, BH, GG, and PN). Unresolved discrepancies were arbitrated by ACS.

A narrative synthesis of the results was conducted, with the relevant study characteristics and intervention details and outcomes presented in the results section. The effect measures reported were those of each study, as well as the results of any statistical analyses. Secondary outcomes reporting usability, side effects, and acceptance of interventions by the participants were also extracted. The significance of the results was established when *P*≤.05. The most relevant follow-up measurements were retrieved from studies to facilitate a more concise synthesis of the results. A meta-analysis was not performed due to the heterogeneity of the study designs and lack of sufficient standardized effect measurements.

### Ethical Considerations

As a systematic review examining publicly accessible secondary data, ethical approval was not necessary for this study. The primary studies considered in this review carried out this process individually.

## Results

### Study Selection

**Figure 1.**
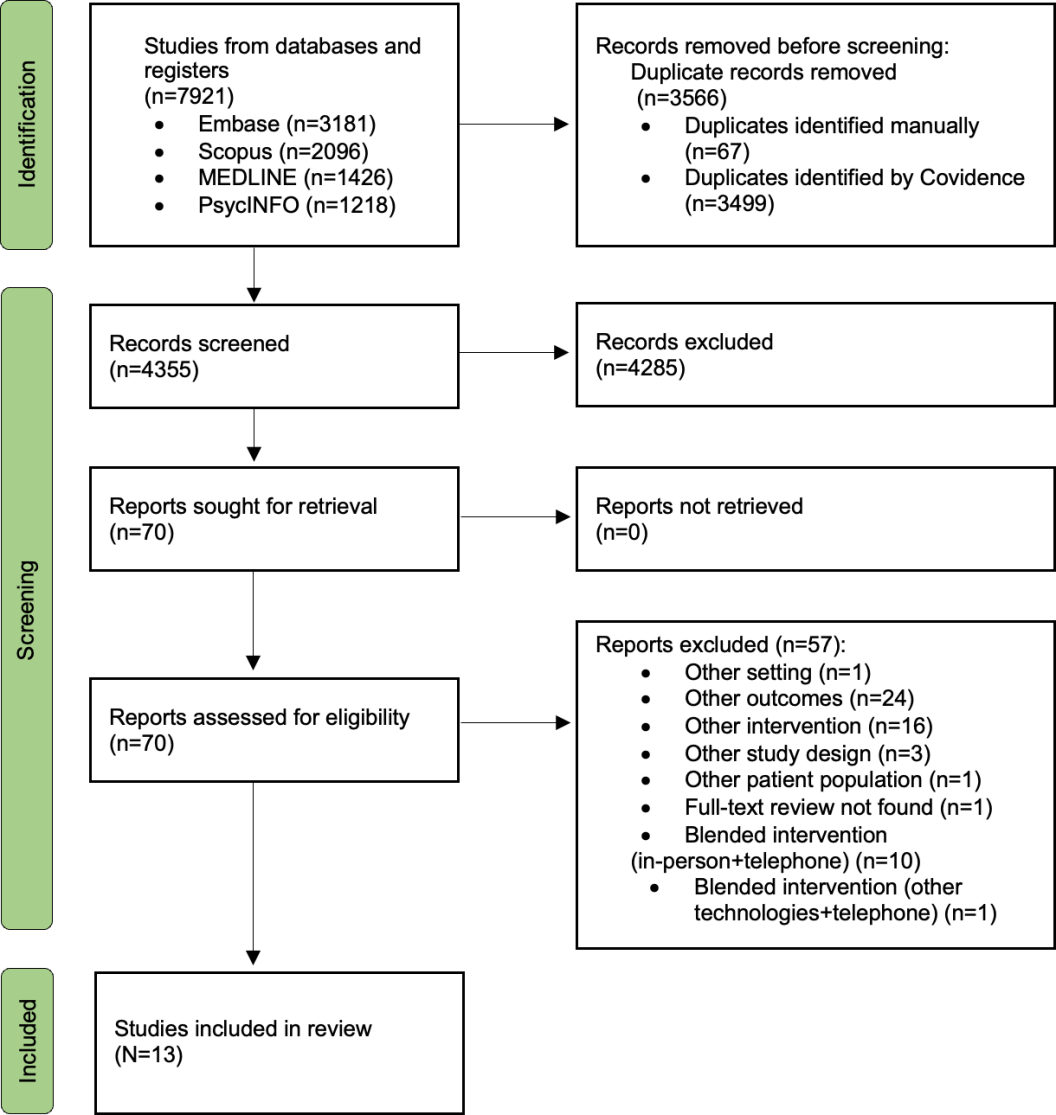
Preferred Reporting Items for Systematic reviews and Meta-Analyses diagram showing the selection process for the studies.

The PRISMA flowchart in Figure 1 summarizes the decision pathway for the final inclusion of the studies in this review. The electronic search of the 4 databases yielded a total of 7921 study records. After removing duplicates, 4355 papers were screened for title and abstract, of which 70 papers were included for full-text review (Figure 1). A total of 13 papers [26-38] were selected for the final analysis.

In Figure 1, other interventions refer to when the type of technology, or application of the technology in the papers did not meet the inclusion criteria of this review. Other blended interventions refer to studies that used telephones alongside other digital technologies, or included an in-person component.

### Study Characteristics

Table 2 shows the characteristics of the studies included. All were conducted in high-income countries: 7 studies [26,27,29,30,36-38] in the United States, 2 studies [31,33] in Germany, 2 [32,34] in Italy, 1 study [35] in Korea, and 1 study [28] in the United Kingdom. Overall, 2 studies [26,27] used the same sample and intervention; therefore, they were merged into one for analysis. The studies were conducted between 2014 and 2024. In total, 8 [26-31,33,34,38] out of the 12 studies [26-38] were randomized controlled trials (RCTs), 2 [32,35] were nonrandomized controlled trials (ie, controlled trials without the process of randomization), and 2 [36,37] were pre-post study interventions with no control group. The sample size ranged from 20 to 440, with a total of 1685 participants.

**Table 2.** Characteristics of the primary studies included for review.

Author and year	Country	Study design	Sample size (n)		Age of participants, mean (SD; range) or median (IQR)		Dementia		Primary outcomes (instrument)
			Intervention group	Control group	Intervention group	Control group	Type	Severity	
Intervention: coaching-based calls									
Bass et al [26], 2014	United States	RCT^a^	202	131	Patients: mean 78.72 (SD 8.64) Caregivers: not specified	Patients: mean 80.32 (SD 6.54) Caregivers: not specified	Not specified	Mild to moderate cognitive impairment	Depression (CESD^b^)
Bass et al [27], 2015	United States	RCT	299	187	Patients: not specified Caregivers: mean 68.57 (SD 12.64)	Patients: not specified Caregivers: mean 71.77 (SD 10.39)	Not specified	Not specified	Behavioral symptoms frequency (0-12) ED^c^ visits Hospital admissions
Cooper et al [28], 2024	United Kingdom	RCT	204	98	Patients: mean 79.7 (SD 8.0) Caregivers: mean 63.1 (SD 12.9)	Patients: mean 80.3 (SD 8.7) Caregivers: mean 64.0 (SD 11.5)	Not specified	Not specified	BPSD^d^ frequency and severity (NPI^e^) Apathy (BDAS^f^)
Intervention: psychosocial and educational support calls									
Mavandadi et al [29], 2017	United States	RCT	38	37	Patients: mean 79.34 (SD 8.58) Caregivers: mean 71.97 (SD 10.92)	Patients: mean 78.54 (SD 8.91) Caregivers: mean 67.94 (SD 12.24)	Not specified	Moderate to severe dementia	BPSD severity (NPI-Q-S^g^) BPSD-related caregiver distress (NPI-Q-D^h^)
Mavandadi et al [30], 2017	United States	RCT	150	290	Patients: not specified Caregivers: mean 64.34 (SD 12.03)	Patients: not specified Caregivers: mean 63.25 (SD 11.89)	Not specified	Moderate to severe dementia	BPSD severity (NPI-Q-S)
Dichter et al [31], 2020	Germany	RCT	19	19	Patients: mean 76.0 (SD 8.0) Caregivers: mean 67.4 (SD 8.1)	Patients: mean 76.3 (SD 8.3) Caregivers: mean 64.1 (SD 10.6)	Not specified	Not specified	Irritability (NPI-Q^i^)
Panerai et al [32], 2021	Italy	Nonrandomized controlled intervention	13	14	Patients: not specified Caregivers: median 65 (IQR 59-69)	Caregivers: 73 (IQR 66-78)	Any type	Mostly mild, some moderate dementia	BPSD frequency and severity (NPI) BPSD-related caregiver distress (NPI-Q-D)
Berwig et al [33], 2022	Germany	RCT	69	72	Patients: not specified Caregivers: mean 73.1 (SD 8.3; 52-85 range)	Patients: not specified Caregivers: mean 74.4 (SD 9.5; 52-90)	Not specified	Not specified	BPSD prevalence
De Stefano et al [34], 2022	Italy	RCT	20		Patients: mean 57.6 (SD 3.8) Caregivers: mean 49 (SD 14.9)	Patients: mean 61.0 (SD 5.0) Caregivers: mean 57.7 (SD 7.7)	Early-onset Alzheimer’s disease (EOAD)	Moderate dementia	BPSD (NPI)
Intervention: online dementia care support platforms									
Park et al [35], 2020	South Korea	Nonrandomized controlled intervention	12	12	Patients: not specified Caregivers: mean 54.50 (SD 3.71)	Caregivers: mean 61.00 (SD 6.42)	Not specified	Not specified	BPSD frequency and severity (K-NPI^j^)
Rodriguez et al [36], 2021	United States	Pre-post study	55	—^k^	Patients: not specified Caregivers: mean 64.96 (SD 10.9)	Caregivers: not applicable	Not specified	Mild to severe dementia	BPSD severity (NPI-Q-S) BPSD-related caregiver distress (NPI-Q-D)
Perales-Puchalt et al [37], 2022	United States	Pre-post study	24	—	Patients: mean 74.9 (SD 12.6) Caregivers: mean 52.6 (SD 13.2)	Patients: not applicable Caregivers: not applicable	Alzheimer disease–related dementias	Not specified	BPSD severity (NPI-Q-S) BPSD-related caregiver distress (NPI-Q-D)
Rodriguez et al [38], 2023	United States	RCT	26	27	Patients: mean 75.9 (SD 10.5) Caregivers: mean 62.5 (SD 13.7)	Patients: mean 77.4 (SD 9.8) Caregivers: mean 63.3 (SD 13.0)	Alzheimer disease	Mostly mild to moderate dementia	BPSD frequency and severity (NPI) BPSD-related caregiver distress (NPI-D^l^)

a RCT: randomized controlled trial.

b CESD: 11-item Center for Epidemiologic Studies Depression Scale.

c ED: Emergency department.

d BPSD: behavioral and psychological symptoms of dementia.

e NPI: Neuropsychiatric Inventory.

f BDAS: Brief Dimensional Apathy Scale.

g NPI-Q-S: Neuropsychiatric Inventory Questionnaire – Severity.

h NPI-Q-D: Neuropsychiatric Inventory Questionnaire–Caregiver Distress.

i NPI-Q: Neuropsychiatric Inventory-Questionnaire

j K-NPI: Korean version of the neuropsychiatric inventory.

k Not applicable.

l NPI-D: Neuropsychiatric Inventory Caregiver Distress Scale.

### Population Characteristics

#### Users of the Intervention

All interventions in this review included informal caregivers as the participants. Overall, 3 studies [26-28,32] involved people living with dementia as part of the intervention delivery. Among them, 2 studies [26-28] coaching calls and 1 study [32] provided psychosocial and educational support to dyads (caregivers and patients with dementia).

When there were challenges for some patients and caregivers to engage in the assessments and they could not be contacted, or when caregivers had physical or cognitive impairments that hindered the consent or intervention process, these groups were often excluded in the studies.

#### Gender

In most studies, approximately 80% (1348) of caregivers were women. Among the studies [26-29,32,37,38] that reported the genders of patients with dementia, on average, from 40% (297) to 60% (446) of the patients were women. Notably, the studies by Bass et al [26,27] specifically targeted veterans and thus predominantly focused on men with dementia. In the same way, one of the studies from Mavandadi et al [29] included mostly male veterans.

#### Ethnicity

Seven studies [26-30,36-38] reported on participants’ ethnicities. Some studies, such as the one from Perales-Puchalt et al [37], focused primarily on Latino caregivers (>60% of the population group of the study). In another study from Rodriguez et al [38], >40% of the sample were from an African American background. However, for the rest of the papers, the proportion of White caregivers and patients in the sample was mostly >75% [26-30,36].

#### Socioeconomic Status

A total of 3 studies [30,33,38] reported that caregivers were from lower-middle-income backgrounds (>60% of participants), with the research from Mavandadi et al [30] only having participants from low-income families. Notably, 2 studies [29,37] assessed financial adequacy, with the paper from Perales-Puchalt et al [37] reporting that participants, on average, experienced mild to moderate financial strain, while in the study from Mavandadi et al [29] >80% of participants had “enough to get along or were financially comfortable.” The rest of the studies [26-28,31,32,34-36] did not specify these population characteristics.

#### Relationship of Caregiver With Patients

All the studies mentioned the relationships of caregivers with the patients. Children of the patients living with dementia were the most common caregivers included, followed by spouses.

### Intervention Characteristics

#### Type of Intervention

Interventions were divided into 3 categories, depending on their mode of delivery and components. Of note, 2 of the studies [26-28] were coaching services delivered through telephone calls for dyads, and 6 [29-34] were telephone calls based on psychosocial and educational support, mostly for caregivers. Finally, 4 [35-38] were online platforms, designed for caregivers to manage dementia care at home. The specific components of the interventions are detailed in Table 3.

**Table 3.** Characteristics of interventions, and most relevant outcomes.

Author and year	Intervention details	Control details	Duration		Frequency of intervention	Effect sizes on primary outcomes	*P* value
			Intervention	Study			
Intervention: coaching-based calls							
Bass et al [26], 2014	A coaching service called PDC^a^, guided by the participants, with care coordinators (health care professionals). Helps find solutions to concerns that are important for veterans and their informal caregivers.	UC^b^ and educational resources from Veterans Affairs.	12 months	12 months	≥1 contact per month	Unstandardized regression coefficient of (β) −0.10 in the CESD^c^ score in the 6-month follow-up in the PDC versus UC group in those with high baseline cognitive	.03
Bass et al [27], 2015	Same intervention as above.	UC and educational resources from Veterans Affairs.	12 months	12 months	≥1 contact per month	BPSD^d^ frequency 6-month follow-up: mean score of 0.22 (SD 0.21) in PDC versus mean score of 0.21 (SD 0.23) in the control group Mean decrease of 32% in hospital admissions on 6-month follow-up between groups with elevated baseline behavioral symptoms^e^ Mean decrease of 28.6% in ED^f^ visits on 6-month follow-up between groups with high 6-month behavioral symptoms	<.05
Cooper et al [28], 2024	Sessions addressing dementia care, coping mechanisms, and goal setting were delivered through nonclinical facilitators, followed by catch-up calls to dyads^g^.	UC and completed goal setting.	12 months	12 months	6-8 sessions over the period of 6 months, followed by follow-up sessions every 2-3 months	NPI^h^ mean change between groups: (β) (95% CI)=0.70 (–5.75 to 7.16) at 12-month follow-up BDAS (emotional): ß=–0.16 (95% CI –0.67 to 0.35) between the groups in 12-month follow-up BDAS (executive): ß=–0.41 (95% CI –0.90 to 0.07) between the groups in 12 month follow-up	—^j^
Intervention: psychosocial and educational support calls							
Mavandadi et al [29], 2017	The first component involves a nurse or social worker establishing and helping with the needs of the caregivers and patients over 6 months. The second is a TEP^k^, which is a caregivers’ group-based telephonic education and psychosocial program.	UC, which involved clinical assessment and signposting caregivers through email to community services for dementia management.	3 months	6 months	≥3 contacts over the period of 3 months	NPI-Q-S^l^ (time x randomization group interaction): β (SE)= –0.32 (0.20) NPI-Q-D^m^ (time x randomization group interaction): β (SE)=–0.68 (0.26)	.11 .01
Mavandadi et al [30], 2017 2	Initial calls with caregivers, and when appropriate, BHPs^n^ signposted them to appropriate resources or services. After caregivers selected 7 modules from an individual TEP, they reviewed a workbook through calls with the BHPs regarding dementia care and problem-solving strategies.	UC, which involved clinical assessment and signposting to services according to caregivers’ and recipients’ needs, with information sent to prescribing clinician.	3 months	6 months	2-3 contacts (TEP took 45-60 minutes per session) over the 3 months	NPI-Q-S (time× intervention group interaction effect): β (SE)=0.01 (0.09)	—
Dichter et al [31], 2020	Moderators, who were psychologists, (1) delivered a preliminary phone call, (2) sent an information booklet before the main intervention to informal caregivers, and (3) moderated 6 group support sessions regarding dementia management and self-care for these carers .	Follow-up assessments of outcomes and continuation of their own organized dementia care and self-care management.	3 months	3 months	1-hour sessions every 2 weeks over the period of 3 months.	NPI-Q^o^ irritability: Difference of 0.41 (95% CI −0.29 to 1.10) in mean scores between 3-month follow-up and baseline score between control and intervention groups	—
Panerai et al [32], 2021	Psychologists delivered group sessions to dyads to discuss different matters regarding dementia care management and individual calls before each group to provide individualized psychological support.	These patients were given a handbook and performed pretest and posttest assessments	4 weeks	4 weeks	10 sessions of 50-60 minutes	NPI frequency intervention versus control group: Spearman rank correlation coefficient (*r)*=–0.45 (medium effect size) NPI severity difference intervention vs control group: *r*=–0.59 (large effect size) NPI-Q-D difference intervention vs control group: *r*=–0.64 (large effect size)	.02
Berwig et al [33], 2022	Carers received a portfolio with aftercare recommendations following a caregiver rehabilitation program. Social workers then moderated 6 aftercare group support sessions. In these sessions, each carer discussed their current application of aftercare recommendations and self-care practices. Then, the moderator gave a presentation on the topic of the session.	Only received portfolio with individualized aftercare recommendations and UC	6 months	12 months	Six 1-hour sessions per month	Risk of falling: difference of 3.4% versus 21.7% increase in prevalence of participants with risk of falling in intervention group versus control group in 6-month follow-up^p^ Rest of the BPSD, 5.4% (tendency to run away) to 35.1% (personality change) increase and 5.1% (bedriddenness) to 48.4% (personality change) increase in 12-month in intervention versus control groups, respectively	.03 —
De Stefano et al [34], 2022	4 telephone support sessions once a week for 1 month delivered by a psychologist. The calls provided emotional support and reflective listening for caregivers.	Only received assessments at different time points	4 weeks	6 months	1-hour session per week	NPI: Increase of mean value from 0.24 (SD 0.11) to 0.32 (SD 0.03) in intervention group compared to decrease of mean value from 0.25 (SD 0.09) to 0.24 (SD 0.08) in control group in 6-month follow-up	.91
Intervention: online dementia care support platforms							
Park et al [35], 2020	CMAP is a comprehensive mobile app program for family caregivers, which includes information about dementia care management, including drugs and nonpharmacological interventions. It also includes caregivers’ coping skills training.	Pretest and posttest assessment, and handbook about dementia care.	3 months	3 months and 2 weeks	Call by a researcher for 5 minutes once a week to answer questions about care, otherwise free use of the app.	For the experimental group, patients’ K-NPI^q^ decreased from mean 0.14 (SD 0.13) before the program to mean 0.11 (SD 0.11) 2 weeks after the termination of the program For the control group, K-NPI from mean 0.16 (SD 0.12) to mean 0.13 (SD 0.14)	—
Rodriguez et al [36], 2021	Informal caregivers attended an orientation training conference to navigate a web-based educational platform developed by experts in the field. Afterward, they had access to a variety of personalized dementia care management education modules.	N/A	1 month	1 month	Access to the platforms throughout the day for 1 month	NPI-Q-S: From mean 0.30 (SD 0.20) to mean 0.27 (SD 0.18); Cohen *d*=0.332 (95% CI 0.06 to –0.61)small effect size NPI-Q-D: From 0.52 (0.32) to 0.47 (0.31). Cohen *d*=0.290 (95% CI 0.02-0.56) small effect size	.02
Perales-Puchalt et al [37], 2022	CuidaTEXT (University of Kansas Medical Center) includes 1-3 automatically sent daily educational messages on dementia and self-care. The participants could also text back to care coaches in the research team whenever they required assistance.	N/A	6 months	6 months	1-3 automatic messages per day related to dementia care	NPI-Q-S: mean 0.45 (SD 0.22) to mean 0.33 (SD 0.15) NPI-Q-D: mean of 0.33 (SD 0.23) to mean 0.20 (SD 0.20)	.004
Rodriguez et al [38], 2023	Psychoeducation and caregiver support. This intervention helps to manage BPSD through individualized live chats with care coaches trained to deliver these interventions.	Individualized care plan with a multidisciplinary team and a care coordinator.	6 months	6 months	Care coaches were sent daily emails and were required to respond to care queries every day.	NPI: –0.02 (95% CI: –0.07 to 0.03) difference between control and treatment groups in 6-month follow-up, compared to –0.003 (95% CI 0.006 to –0.0006) at baseline NPI-D^r^: –0.005 (95% CI –0.07 to 0.05) difference between groups at 6 months, compared to –0.002 (95% CI –0.01 to 0.005) at baseline	.43

a PDC: Partners in Dementia Care.

b UC: Usual care.

c CESD: 11-item Center for Epidemiologic Studies Depression Scale.

d BPSD: behavioral and psychological symptoms of dementia.

e These numbers were taken on the assumption that hospitalizations and ED visits were related to BPSD because they were patients who had high baseline behavioral symptoms (for hospital admissions) or 6-month behavioral symptoms (for ED visits).

f ED: Emergency department.

g Dyads: Patients with dementia and informal caregivers.

h NPI: Neuropsychiatric Inventory.

i BDAS: Brief Dimensional Apathy Scale.

j Nonsignificant results (*P*>.05).

k TEP: Telehealth educational program.

l NPI-Q-S: Neuropsychiatric Inventory Questionnaire – Severity.

m NPI-Q-D: Neuropsychiatric Inventory Questionnaire – Caregiver Distress.

n BHP: behavioral health provider.

o NPI-Q: Neuropsychiatric Inventory–Questionnaire.

p The only BPSD component that demonstrated a statistically significant difference.

q K-NPI: Korean version of the neuropsychiatric inventory.

r NPI-D: Neuropsychiatric Inventory Caregiver Distress Scale.

#### Intervention Delivery

Four interventions [29,31-33] were delivered as part of a group setting to either caregivers or dyads, while the rest of the interventions were delivered to the participants individually.

None of the studies, apart from the ones that were based on online platforms, specified the type of telephone used in the treatments. Three of the interventions [35,36,38] delivered through digital platforms required a smartphone, and one [37] at least a mobile phone as they contained features such as apps, touchscreens, and text messages. These interventions did not include a telephone call component, but some involved live messaging chats with care providers [37,38]. Nevertheless, 8 [26-34] out of the 12 studies’ [26-38] interventions were based on calls; therefore, it can be assumed that landlines could be used too to deliver those services. Finally, no significant difficulties were reported in the use of telephones by the providers, patients, or caregivers who participated in the studies.

#### Duration and Frequency of Interventions

A total of 2 interventions [26-28] lasted for 12 months. Among them, 3 [33,37,38] lasted for 6 months, 4 [29-31,35] lasted for 3 months, and 3 [32,34,36] lasted for less than 1 month.

Among the interventions that specified session length, telephone calls lasted between 45 minutes and 1 hour. In the interventions that were based on telephone calls, patients were generally contacted more than once a month, and in some studies, even more than once every week [31,34].

Finally, regarding online platforms, in all interventions, patients were able to use the service every day, with 2 [37,38] of these 4 interventions [35-38] having direct access to people delivering care.

### Effectiveness of Telephone Interventions

#### Measurement of Outcomes

The approach used to measure the primary outcomes varied depending on the specific methodology used in the studies. Validated measurements to evaluate the change in BPSD in patients living with dementia included standardized scales, such as the Neuropsychiatric Inventory Questionnaire (NPI-Q-S), which measures the severity of neuropsychiatric symptoms. Other studies measured specific symptoms, such as depression, individually.

BPSD-related caregiver burden was measured by BPSD-related caregiver distress, with scales, such as the Neuropsychiatric Inventory Questionnaire – Caregiver Distress (NPI-Q-D). Finally, in the study reporting on BPSD-related hospitalizations, the authors measured changes in the number of hospital or emergency department (ED) admissions attributable to these symptoms [27]. Secondary outcomes included acceptability and usability of the interventions and any reported side effects.

#### BPSD in Care Recipients

Overall, 9 out of 12 studies [26-29,32,33,35-38] reported positive effects of telephone interventions in BPSD in patients with dementia. In terms of individual symptoms, 1 study [26,27] reported a statistically significant effect of the telephone interventions on depression symptoms (β=−0.1; *P*=.03) [26], while another study highlighted a reduced risk of falling compared to the control group [33]. However, these technologies did not lead to a significant decrease in apathy [28] or a noticeable increase in irritability [31].

Of studies analyzing all BPSD symptoms, 3 studies [32,36,37] demonstrated a statistically significant effect on the NPI-Q-S score or BPSD frequency and severity. For example, the study from Panerai et al [32] demonstrated a large effect size in BPSD severity ( Spearman rank correlation coefficient, *r*=−0.59) . However, 5 studies [27,29,30,35,38] found no statistically significant improvement on BPSD incidence, severity, and frequency. Finally, 3 studies [28,30,34] showed an increase in the NPI (Neuropsychiatric Inventory) score, but these changes were not statistically significant, with the study from De Stefano et al [34] showing an mean increase of NPI of 0.08 in the treatment group, as seen in Table 3.

#### BPSD-Related Informal Caregiver Burden

Of the 5 studies [29,32,36-38] that analyzed BPSD-related caregiver burden, 4 [29,32,36,37] demonstrated a statistically significant decrease in BPSD-related caregiver distress. Indeed, the RCT from Mavandadi et al [29] showed that telephone calls caused a significant decrease in BPSD-related distress in the 6-month follow-up (*P*=.01). However, Rodriguez et al [38] found that their internet-based app led to a nonsignificant decrease in Neuropsychiatric Inventory Caregiver Distress Scale (NPI-D) [38].

#### BPSD-Related Hospitalizations of Care Recipients

The study conducted by Bass et al [26,27] reported a statistically significant reduction in BPSD-related hospital admissions and ED visits. This showed a decrease of 32% and 28.6% in hospital and ED admissions, respectively, 6 months after the coaching service started. However, no statistically significant differences were seen in the 12 month follow-up. This data were taken from the national patient care databases.

#### Secondary Outcomes

In total, 4 studies [28,36-38] reported on 3 of the secondary outcomes specified in the methods: acceptability, side effects, and usability. Perales-Puchalt et al [37] demonstrated a mean score of 96% (SD 9.7%) on usability of the dementia care support delivered via text messaging and 75% satisfaction with the intervention. These findings were similar to Rodriguez et al [38], with an app usability mean of 72.5% (95% CI 64.1‐81.2) and a user acceptance of 85%‐90%. Cooper et al [28] reported a 10% withdrawal of dyads from the intervention; however, the specific reasons were not reported.

#### Risk of Bias

The risk of bias was calculated using the NHLBI (National Heart, Lung, and Blood Institute) quality assessment tool. Studies that were randomized or nonrandomized controlled trials were considered to be of good quality if 10 out of 14 or more questions in the assessment tool were answered “yes.” Similarly, pre-post intervention studies were deemed good quality if 9 out of 12 questions were answered ’“yes.” A total of 3 studies [28,31,38] fit this criterion. Approximately 8 [26,27,29,30,32,33,35-37] out of the 12 studies were of fair quality [26-38], as they fulfilled >5/14 and 4/12 of the criteria in the questionnaire. Overall, 1 out of the 12 studies was poor quality [34]. The detailed risk of bias assessment of each study can be found in Multimedia Appendix 3.

Table 3 represents a summary of the main characteristics of the interventions and the main follow-up outcomes for each study. Finally, Table 4 shows a summary of the overall effect of interventions according to each outcome and their associated GRADE (Grading of Recommendations Assessment, Development and Evaluation) score.

A detailed analysis and justification of each GRADE score can be found on the Multimedia Appendix 4.

**Table 4.** Summary of the effect of interventions on key outcomes and their GRADE (Grading of Recommendations Assessment, Development and Evaluation) certainty score.

Outcome	Overall effect of interventions	Sample size	GRADE^a^ certainty
BPSD^b^	Positive	1685	Low
BPSD-related caregiver burden	Positive	234	Low
BPSD-related hospitalizations^c^	N/A^d^	486	N/A

a GRADE: Grading of Recommendations Assessment, Development and Evaluation.

b BPSD: behavioral and psychological symptoms.

c Only one study measured this outcome; therefore, it was not possible to state the overall effect of the interventions nor the GRADE certainty in this outcome.

d Not applicable.

## Discussion

### Summary of Main Findings

This systematic review identified 12 studies [26-38] from 5 high-income countries that examined the effectiveness of different types of telephone interventions. Overall, 3 categories of telephone interventions were demonstrated to improve BPSD and BPSD-related informal caregiver burden. Only 1 [27] study reported on BPSD-related hospitalizations, indicating a statistically significant decrease in ED and hospital admissions. In total, 4 [28,36-38] of the 12 studies also reported on secondary outcomes, revealing good usability, acceptability, and no specific adverse effects reported by participants.

### Comparison With Previous Literature

#### BPSD Reduction

While most studies in this review reported improvements in BPSD-related symptoms, it is not possible to draw definitive conclusions about the overall effectiveness of the interventions due to the heterogeneity in their frequency, duration, and components. Notably, all studies in this review showing statistically significant improvements were deemed fair quality. The GRADE certainty for this outcome was rated as low, primarily due to the risk of bias in the studies and imprecision of results.

However, interventions that were more personalized, involving both patients with dementia and their informal caregivers in developing the treatment plan, appeared to be more effective, at least for a period of 6 months. For instance, the studies from Bass et al [26,27] and Panerai et al [32] showed a statistically significant improvement in the symptoms over the periods of 6 months and 1 month, respectively. These studies collaborated with caregivers and patients with dementia in the development of the treatment plan.

In contrast, interventions that provided more generalized guidance for dyads appeared to be less effective in reducing these symptoms, which aligns with the findings from other existing umbrella or systematic reviews [39,40]. These studies argued that involving patients with dementia and caregivers in the design of the interventions could prove a more effective and acceptable approach in the management of BPSD at home. This may be particularly important because BPSD symptoms are very complex, and the specific needs and triggers of patients are different, as are the coping strategies, cultural context, and caregivers’ knowledge [41,42].

#### BPSD-Related Caregiver Burden

The generally positive significant outcomes of these interventions on BPSD-related caregiver burden may be attributed to the well-established effectiveness of psychosocial and educational dementia care support for dyads in alleviating caregivers’ burden [4]. Informal caregivers often experience significant burden partially due to a lack of information on managing BPSD at home, insufficient psychosocial support, and strategies to prioritize their self-care [6,8]. All interventions identified in this review involved strategies to address these support gaps, which may explain their positive impact.

These findings are consistent with broader evidence suggesting that technology-delivered support, including telephone-based interventions, can reduce the burden and enhance the well-being of informal caregivers of people with dementia [43,44].

It is also important to note the potential of small group interventions to be beneficial for caregivers. Two studies [29,32] in this review demonstrated significant benefits of delivering small group interventions. This aligns with previous literature supporting group-based interventions as a well-accepted and potentially effective approach, likely due to their capacity to reduce social isolation and provide emotional support among caregivers facing similar challenges with dementia care [12,45].

However, it is important to note that the GRADE certainty of the evidence for this outcome was rated as low due to the variety of methodological designs and risk of bias in the studies. Thereby, these conclusions have to be interpreted with caution, and further research is needed to confirm these findings.

#### BPSD-Related Hospital Admissions

BPSD-related hospitalizations are common, but strategies on prevention remain scarce. However, the study conducted by Bass et al [26,27] could serve as an example of how structured telephone interventions might help address this issue. As inadequate support and management of BPSD in the community increases the risk of hospitalizations, telephone-based interventions could serve as a tool to help caregivers manage challenging behaviors at home, potentially reducing hospitalizations. However, current evidence on the most effective interventions to decrease these health care admissions remains inconclusive [46,47].

### Strengths and Limitations

To the best of our knowledge, this is the first systematic review summarizing the effectiveness of telephone interventions in the management of BPSD by informal caregivers and patients at home. It is also the first to assess the impact of these technologies on key stakeholders—individuals living with dementia and informal caregivers—on health care resource usage. In the same way, the cost and relative simplicity of the interventions, compared to other more technologically complex approaches, could further increase the feasibility of implementation in low-resource settings and more constrained health care systems [14,48].

However, it must be considered that BPSD and BPSD-related caregiver burden are highly complex and multifactorial. While the NPI-Q and NPI-Q-D are standardized and widely used scales, they do not entirely capture the impact and change of individual symptoms or the complexity of factors that affect caregiver burden, respectively. For instance, each symptom can respond to treatment differently [49]. Similarly, some symptoms can cause more burden for caregivers than others; for example, aggressions may cause more caregiver distress than others, such as appetite changes. In the same way, there are a variety of factors that affect caregivers' burden, such as cognitive symptoms or level of social support available [1,50]

Therefore, while the NPI-Q-D provides valuable insight, it captures just some part of this experienced burden.

This review focused on quantitative measurements; therefore, a qualitative review was not feasible. However, understanding and interpreting the extent to which the different types of interventions are effective would be key to ensure that they have a clinically significant impact on complex phenomena, such as BPSD and related burdens [51,52]. This is especially important with scales such as NPI-Q or NPI-Q-D, for which currently there is no established threshold to define a clinically meaningful change [53].

Given the complexity of technology use in dementia care, qualitative findings could help enhance the interpretation of quantitative outcomes and support the development of more personalized interventions. It can also aid in identifying specific barriers or difficulties dyads may experience when using these technologies, especially those with limited health and digital literacy levels, disabilities, or language difficulties [54].

### Implications of Findings for Future Practice and Research

The improvements that telephone interventions demonstrated in BPSD-related caregiver burden and BPSD in care recipients, as well as the positive usability and acceptability scores, support the potential use of telephone interventions as a tool for managing these symptoms in home settings. In addition, most studies included in this review are RCTs, reducing the likelihood of confounding factors impacting the effectiveness of the various interventions, such as the dyads' ages or the relationship of caregivers with patients living with dementia.[13,55]

These interventions also have the potential to enable continuity of care for many families, given the flexibility and accessibility these treatments provide for patients with dementia, informal caregivers, and health care professionals. These services may also complement existing in-person support and contribute to a more holistic and equitable model of dementia care [56].

However, this review also highlights important gaps that require further exploration.

First, all studies from this review emanated from high-income countries. As such, it is crucial to interpret these findings according to the context of the health care system within each place, as factors, such as health and social care professionals’ knowledge about dementia, staff and resource availability, and culture can play a key role in the effectiveness of these interventions. [2,57]

More studies focusing on LMICs are needed to ensure the applicability of findings in diverse health care settings. One way in which this could be achieved is through intercountry collaborations, which could support research centers in these regions in having necessary funding and resources to conduct these trials. These efforts could be grounded in strong community engagement, involving dyads, local professionals, and institutions from LMICs. [58,59] Developing telephone co-interventions with stakeholders in these communities will be key to ensuring they are accessible, acceptable, and effective in different contexts from those in these studies [60] In addition, future systematic reviews could incorporate studies conducted in LMICs by expanding searches or the inclusion criteria of the interventions, to include supplementary databases.

The long-term effectiveness of these interventions warrants further research, particularly given the importance of continuity of care in dementia and the current fragmented nature of dementia care.[61,62] More RCTs with longer follow-up periods will be key to evaluating the sustainability and feasibility of these interventions at scale. Similarly, cost-effectiveness analyses are needed to support future policy recommendations. This could be achieved by tracking changes in hospital admission rates and associated costs when dyads use these tools and transparently illustrating the costs of these interventions.[63]

Finally, telephone interventions to manage BPSD in the community have the potential to help achieve the "Global Action Plan on the Public Health Response to Dementia" [21], which aims for at least 75% of families worldwide to receive dementia training and support. They can also become an essential component of national digital transformation programs, such as the NHS long-term plan [22]. However, it is important to tailor the interventions according to the needs, resources, and health care staff available in each region. For example, in lower-income settings, the telephone platforms could prove more helpful because they are asynchronous. [64] Patients and caregivers would not require internet access or the immediate availability of a health care professional in order to benefit from these interventions. Investing in large-scale research on these interventions could be highly efficient in leveraging these mainstream technologies within health care systems to achieve more equitable dementia care both within and across countries.[65]

### Conclusions

Telephone interventions delivered through psychosocial and educational calls and online platforms are promising tools for reducing BPSD-related caregiver burden. Personalized telephone interventions, including patients’ and informal caregivers’ input in the treatment plan, may improve community-dwelling patients’ BPSD severity and frequency. However, the certainty of evidence for both outcomes was low; therefore, these findings should be interpreted with caution. Further high-quality research of the interventions in low- and middle-income countries with longer follow-up periods, including cost-effectiveness analyses and greater consistency in intervention design and outcome measurement, is required to establish the global generalizability of these interventions and inform future practice.

## Supplementary material

10.2196/77233 Multimedia Appendix 1 Full search strategy for MEDLINE, SCOPUS, Embase, and Psycinfo.

10.2196/77233 Multimedia Appendix 2 Population, Intervention, Comparison, Outcomes, and Study Design table with in-depth rationale of selection criteria choices.

10.2196/77233 Multimedia Appendix 3 Risk of bias using the National Heart, Lung, and Blood Institute quality assessment tool.

10.2196/77233 Multimedia Appendix 4 Grade certainty table for each outcome of interest.

10.2196/77233 Checklist 1 PRISMA 2020 checklist.

## Abbreviations

BPSD: behavioral and psychological symptoms of dementia
ED: emergency department
GRADE: Grading of Recommendations Assessment, Development, and Evaluation
K-NPI: Korean version of the neuropsychiatric inventory
LMIC: low- and middle-income country
NHLBI: National Heart, Lung, and Blood Institute
NHS: National Health Service
NPI: Neuropsychiatric inventory (NPI-D for Caregiver distress)
NPI-Q: Neuropsychiatric Inventory questionnaire
NPI-Q-S: Neuropsychiatric Inventory Questionnaire-Severity
PICOS: Population, Intervention, Comparison, Outcomes, and Study Design
PRISMA: Preferred Reporting Items for Systematic reviews and Meta-Analyses
PROSPERO: International Prospective Register of Systematic Reviews
RCT: randomized controlled trial
WHO: World Health Organization
