# Supplementary material for: Effectiveness of Telephone Interventions for the Management of Behavioral and Psychological Symptoms of Dementia in the Community: Systematic Review
Source: J Med Internet Res. 2025 Oct 20;27:e77233. doi: 10.2196/77233 (PMC12536943; doi:10.2196/77233)
Supplement: Multimedia Appendix 1 [file jmir-v27-e77233-s001.docx]

**Supplementary file 1:**

**Full search strategy for MEDLINE, SCOPUS, Embase, and Psycinfo.**

Database: *MEDLINE (OVID)*

Search Strategy:

1 dement*.mp. (172192)

2 exp Dementia/ (212833)

3 lewy body disease*.mp. (5515)

4 aggression*.mp. (55843)

5 aggressive*.mp. (237660)

6 appetite.mp. or Appetite/ (41227)

7 Psychomotor Agitation/ or agitation*.mp. (23530)

8 apathy.mp. or Apathy/ (6944)

9 disinhibition.mp. (7688)

10 pacing.mp. (46728)

11 exp Sleep Wake Disorders/ or sleep problem*.mp. (116431)

12 sleep disorder*.mp. (30754)

13 wandering.mp. (4915)

14 yelling.mp. (191)

15 anxiety.mp. or Anxiety/ (326309)

16 delusion*.mp. (15951)

17 depression.mp. (513352)

18 hallucination*.mp. or Hallucinations/ (21233)

19 Mania/ or mania*.mp. (13091)

20 psychosis.mp. (48073)

21 BPSD.mp. (1463)

22 exp Cell Phone/ or exp Telemedicine/ or phone.mp. or Telephone/ (105433)

23 iphone.mp. (1227)

24 android.mp. (4057)

25 telephone.mp. (76417)

26 e health.mp. (4471)

27 ehealth.mp. (7663)

28 m health.mp. (979)

29 mhealth.mp. (10305)

30 e consult*.mp. (377)

31 econsult*.mp. (248)

32 online consult*.mp. (411)

33 remote consult*.mp. (6685)

34 social media.mp. (38558)

35 exp Internet/ (99843)

36 internet.mp. (135265)

37 multimedia.mp. or Multimedia/ (7330)

38 zoom.mp. (3999)

39 telemedicine.mp. (51957)

40 mobile app*.mp. (19172)

41 Mobile Applications/ or smartphone.mp. (31866)

42 neuropsychiatric.mp. (42851)

43 behavio?r*.mp. (2075994)

44 alzheimer*.mp. (211827)

45 insomnia.mp. (31369)

46 depressiv*.mp. (222970)

47 anxious.mp. (21210)

48 psychological*.mp. (691084)

49 neuropsychiatry.mp. or Neuropsychiatry/ (3827)

50 1 or 2 or 3 or 44 (337769)

51 4 or 5 or 6 or 7 or 8 or 9 or 10 or 11 or 12 or 13 or 14 or 15 or 16 or 17 or 18 or 19 or 20 or 21 or 42 or 43 or 45 or 46 or 47 or 48 or 49 (3494789)

52 22 or 23 or 24 or 25 or 26 or 27 or 28 or 29 or 30 or 31 or 32 or 33 or 34 or 35 or 36 or 37 or 38 or 39 or 40 or 41 (359879)

53 50 and 51 and 52 (1426)

Database: *SCOPUS (OVID)*

Search strategy:

( TITLE-ABS-KEY ( dement* OR alzheimer* OR "lewy body disease" OR huntington OR cadasil OR "corticobasal syndrome" OR creutzfeldt-jakob OR "normal pressure hydrocephalus" OR "progressive supranuclear palsy" OR "HIV-associated neurocognitive disorder" ) AND TITLE-ABS-KEY ( behaviour* OR aggression* OR aggressive* OR appetite OR agitation* OR apathy OR disinhibition OR pacing OR "sleep problem" OR "sleep disorder" OR wandering OR yelling OR psychological* OR anxiety OR anxious OR delusion OR depression OR depressiv* OR hallucination* OR insomnia* OR mania* OR psychosis OR bpsd OR neuropsychiatric OR neuropsychiatry ) AND TITLE-ABS-KEY ( phone OR iphone OR android OR telephone OR "ehealth" OR "mhealth" OR "econsult" OR "online consult" OR "remote consult" OR "social media" OR internet OR multimedia OR zoom OR telemedicine OR telehealth OR "mobile app" OR smartphone )

Database: *Embase Classic+Embase* *(OVID)*

Search Strategy:

1 exp dementia/ (467658)

2 dement*.mp. (273380)

3 alzheimer*.mp. [mp=title, abstract, heading word, drug trade name, original title, device manufacturer, drug manufacturer, device trade name, keyword heading word, floating subheading word, candidate term word] (317250)

4 lewy body disease*.mp. (12951)

5 exp aggression/ or aggression.mp. (155950)

6 aggressive*.mp. (360638)

7 decreased appetite/ or appetite/ or "loss of appetite"/ or appetite.mp. (94802)

8 agitation.mp. or agitation/ (52120)

9 apathy.mp. or apathy/ (16341)

10 disinhibition.mp. or behavioral disinhibition/ (11308)

11 pacing.mp. (69492)

12 exp insomnia/ or exp sleep disorder/ or sleep problem*.mp. or insomnia.mp. or sleep disorder*.mp. (325255)

13 wandering.mp. (6277)

14 yelling.mp. (287)

15 anxiety/ or anxiety.mp. (535780)

16 anxious.mp. [mp=title, abstract, heading word, drug trade name, original title, device manufacturer, drug manufacturer, device trade name, keyword heading word, floating subheading word, candidate term word] (31057)

17 delusion/ or delusion*.mp. (32517)

18 depression.mp. or depression/ (912952)

19 depressiv*.mp. (226122)

20 hallucination*.mp. or hallucination/ (53038)

21 mania*.mp. or mania/ (32861)

22 psychosis/ or psychosis.mp. (172485)

23 psychological*.mp. (924994)

24 neuropsychiatric inventory/ or neuropsychiatric*.mp. or neuropsychiatry.mp. [mp=title, abstract, heading word, drug trade name, original title, device manufacturer, drug manufacturer, device trade name, keyword heading word, floating subheading word, candidate term word] (73863)

25 behavio?r*.mp. (2581258)

26 BPSD.mp. (2270)

27 1 or 2 or 3 or 4 (549531)

28 5 or 6 or 7 or 8 or 9 or 10 or 11 or 12 or 13 or 14 or 15 or 16 or 17 or 18 or 19 or 20 or 21 or 22 or 23 or 24 or 25 or 26 (4775863)

29 phone.mp. (73646)

30 telephone.mp. (119302)

31 telemedicine.mp. (57291)

32 iphone.mp. (2642)

33 android.mp. (6377)

34 e health.mp. or exp telehealth/ (95864)

35 ehealth.mp. (7691)

36 m health.mp. (1240)

37 mhealth.mp. (9372)

38 e consult*.mp. (648)

39 econsult*.mp. (412)

40 online consult*.mp. (635)

41 exp teleconsultation/ or remote consult*.mp. (17272)

42 social media.mp. or social media/ (62055)

43 Internet/ or internet.mp. (171287)

44 multimedia.mp. or multimedia/ (10728)

45 zoom.mp. (7251)

46 exp mobile application/ or mobile app*.mp. (30265)

47 smartphone.mp. (37260)

48 exp mobile phone/ (50680)

49 29 or 30 or 31 or 32 or 33 or 34 or 35 or 36 or 37 or 38 or 39 or 40 or 41 or 42 or 43 or 44 or 45 or 46 or 47 or 48 (524689)

50 27 and 28 and 49 (3172)

Database: *APA PsycInfo* *(OVID)*

Search Strategy:

1 dement*.mp. (93576)

2 exp Dementia/ (95025)

3 semantic dementia/ (2848)

4 alzheimer*.mp. (79562)

5 exp Aggressive Behavior/ or aggression*.mp. (260731)

6 aggressive*.mp. (71776)

7 Appetite/ or appetite.mp. (11607)

8 agitation.mp. or Agitation/ (8909)

9 apathy.mp. or Apathy/ (6156)

10 disinhibition.mp. (6231)

11 pacing.mp. (2343)

12 insomnia.mp. or exp Insomnia/ (17596)

13 exp Sleep Wake Disorders/ or sleep problem*.mp. (28280)

14 sleep disorder*.mp. (10930)

15 wandering.mp. (2951)

16 yelling.mp. (281)

17 Anxiety/ or anxiety.mp. (297762)

18 anxious.mp. (25412)

19 Delusions/ or delusion*.mp. (18244)

20 Major Depression/ or depression.mp. (399770)

21 depressiv*.mp. (170630)

22 hallucination*.mp. or Hallucinations/ (18094)

23 Mania/ or mania*.mp. (18547)

24 psychosis.mp. or Psychosis/ (62552)

25 psychological*.mp. (603305)

26 Neuropsychiatry/ or neuropsychiatric.mp. (40023)

27 neuropsychiatry.mp. (8823)

28 behavio?r*.mp. (1525226)

29 BPSD.mp. (1005)

30 5 or 6 or 7 or 8 or 9 or 10 or 11 or 12 or 13 or 14 or 15 or 16 or 17 or 18 or 19 or 20 or 21 or 22 or 23 or 24 or 25 or 26 or 27 or 28 or 29 (2426915)

31 phone.mp. (14518)

32 telephone.mp. (30400)

33 telemedicine.mp. or exp Telemedicine/ (16786)

34 iphone.mp. or exp Mobile Devices/ (12281)

35 android.mp. (691)

36 e health.mp. or Electronic Health Services/ (2506)

37 ehealth.mp. (1860)

38 exp Telemedicine/ or Mobile Health/ or m health.mp. (16852)

39 mhealth.mp. (2200)

40 e consult*.mp. (71)

41 econsult*.mp. (28)

42 online consult*.mp. (107)

43 remote consult*.mp. (847)

44 exp Social Media/ or social media.mp. (33723)

45 internet.mp. or exp Internet/ (69045)

46 multimedia.mp. or Multimedia/ (6476)

47 zoom.mp. (1829)

48 Mobile Applications/ or mobile app*.mp. (5246)

49 smartphone.mp. (6528)

50 31 or 32 or 33 or 34 or 35 or 36 or 37 or 38 or 39 or 40 or 41 or 42 or 43 or 44 or 45 or 46 or 47 or 48 or 49 (164745)

51 1 or 2 or 3 or 4 (132933)

52 30 and 50 and 51 (1218)
