# Supplementary material for: Effectiveness of Telephone Interventions for the Management of Behavioral and Psychological Symptoms of Dementia in the Community: Systematic Review
Source: J Med Internet Res. 2025 Oct 20;27:e77233. doi: 10.2196/77233 (PMC12536943; doi:10.2196/77233)
Supplement: Multimedia Appendix 2 [file jmir-v27-e77233-s002.docx]

**Supplementary file 2.**

***Table 5:* PICOS table with in-depth rationale of selection criteria choices.**

| **PICOS term** | **Choice** | **Explanation** |  |
| --- | --- | --- | --- |
| **Population** | | Community-dwelling people with dementia, and their informal caregivers | Most patients with dementia live at home in the U.K. and worldwide, and they can be more difficult to reach because they are not specifically in care or nursing homes. Some of them also live in remote areas, making it more difficult for them to attend in-person appointments. It is also widely-known that the majority of patients want to remain at home as long as possible. The findings of this study would therefore be more applicable as well to families from a lower-socioeconomic status, and in LMICs, as in those countries and settings most care is provided at home, often by informal carers. Furthermore, informal caregivers often struggle with biopsychosocial issues such as depression, anxiety, and financial difficulties as a consecuence of the caregiving activities, and the emotional impact of the grief caused by the disease. Lastly, the well-being of community-dwelling people with dementia, and their informal caregivers is very closely linked and interdependent, to the extent that it is able to influence the frequency of hospitalisations in these patients. |
| **Intervention** | | Telephone-based interventions for people with dementia, and their informal caregivers | These would include the following interventions:  - Mobile applications: including social media, and m-health platforms.  - Telemedicine: these would include e-consults, text messages, and online interventions such as remote psychosocial support.  However, those primary studies which research the impact of mobile-based interventions which require wearables, or other digital technologies, will be excluded as they would increase the cost and accessibility of the interventions.  These have been selected as the intervention firstly, as previous systematic reviews have demonstrated the potential of these technologies to improve dementia care. With over half of the population owns a smartphone. Another advantage of these interventions is they do not always require a broadband connection to work |
| **Control** | | Before the intervention, compared to a control group, or compared to the standard of care (SOC) | The evidence is aimed to be quantitative so the control groups will vary depending on the study design.  Some studies found compared the intervention in the population groups. |
| **Outcome** | | Management of behavioural and psychological symptoms of dementia in the community. | The management of BPSD in the community will be accessed in by:   - Analysing the improvement of BPSD of community-dwelling older adults. This would be done through standardised measurements such as geriatric depression scale (GDS) or the neuropsychiatric inventory questionnaire (NPI). - Analysing the improvement in BPSD-related informal caregiver distress. This will be done through widely-utilised formal tools such as the Neuropsychiatry questionnaire (NPI-Q-S). - Quantifying the change in the number of BPSD-related hospitalisations or admissions.   These were chosen as main outcomes of the studies because of their relevance in dementia care:  BPSD is largerly associated with a decrease in quality of life both in dementia patients and their informal carers, as well as more frequent hospitalisations. Furthermore, it is estimated to affect 97% of patients with dementia in the community. Therefore, measuring the effects of mobile-based interventions in the management of these symptoms by key stakeholders in the community can help ensure the relevance of the findings of the systematic review in public health policy in different settings. |
| **Study design** | | Quantitative/mixed-method studies | These will range from observational studies to randomized controlled trials. The type of studies have not been limited due to the novelty this area of research is, and the limited number of primary studies found on this topic. Furthermore, the variability of study designs could help to ensure applicability of findings in different settings as well, not only in highly controlled ones. Qualitative studies were excluded due to the limited time frame of this systematic review.  We will most likely probably exclude the studies that are blended interventions, as we want to analyse the effects of only online interventions. However, this is to be discussed once the first part of the screening has been done. |
