# Supplementary material for: Effectiveness of Telephone Interventions for the Management of Behavioral and Psychological Symptoms of Dementia in the Community: Systematic Review
Source: J Med Internet Res. 2025 Oct 20;27:e77233. doi: 10.2196/77233 (PMC12536943; doi:10.2196/77233)
Supplement: Multimedia Appendix 3 [file jmir-v27-e77233-s003.docx]

**Supplementary file 3.**

***Table 6:* Risk of bias using the NHBLI quality assessment tool**

| **Author, year** | **Study design** | **Q1** | **Q2** | **Q3** | **Q4** | **Q5** | **Q6** | **Q7** | **Q8** | **Q9** | **Q10** | **Q11** | **Q12** | **Q13** | **Q14** |
| --- | --- | --- | --- | --- | --- | --- | --- | --- | --- | --- | --- | --- | --- | --- | --- |
| M. Bass et al., 2015 | RCT | Y | N/R | N/R | N/R | N/R | Y | N | Y | Y | Y | Y | Y | Y | N |
| Park et al., 2020 | Non-randomized controlled intervention | N | N/A | N/A | N/A | N/A | Y | Y | Y | NR | Y | Y | N | Y | N |
| Perales-Puchalt et al., 2022 | Pre-post test intervention | Y | Y | C/D | N/R | N | Y | Y | N | Y | Y | N | N/A | N/A | N/A |
| Mavandadi et al., 2017 | RCT | Y | Y | NR | N | N | Y | N | N | NR | NR | Y | N | Y | Y |
| Rodriguez et al., 2023 | RCT | Y | Y | Y | Y | NR | Y | Y | Y | Y | Y | Y | N | Y | Y |
| Mavandadi et al., 2017 2 | RCT | N | N | NR | N | NR | Y | N | Y | N | Y | Y | Y | Y | Y |
| Dichter et al., 2020 | RCT | Y | Y | Y | N | Y | Y | Y | Y | CD | N | N | Y | Y | Y |
| Berwig, 2022 | RCT | Y | Y | NR | N | NR | Y | N | NR | Y | Y | N | Y | Y | N |
| Panerai et al., 2021 | Non-randomised controlled intervention | N | N/A | N/A | NR | Y | Y | Y | Y | NR | Y | Y | N | Y | N/A |
| Rodriguez, 2021 | Pre-post test intervention | Y | Y | N | N | NR | Y | Y | NR | Y | Y | N/A | N/A | N/A | N/A |
| M Bass et.al, 2014 | RCT | Y | NR | NR | NR | NR | N | N | Y | N | Y | Y | Y | Y | Y |
| De Stefano et al.,2022 | RCT | Y | NR | NR | NR | NR | Y | NR | NR | NR | Y | Y | N | N | NR |
| Cooper et al., 2024 | RCT | Y | Y | Y | N | Y | Y | Y | Y | Y | Y | Y | Y | Y | Y |

Abbreviations: Y: Reported, and present in the study; N: Reported, but not present in the study; N/A: Not applicable for the specifc study design; NR: Not reported in the study
