# Supplementary material for: Effectiveness of Telephone Interventions for the Management of Behavioral and Psychological Symptoms of Dementia in the Community: Systematic Review
Source: J Med Internet Res. 2025 Oct 20;27:e77233. doi: 10.2196/77233 (PMC12536943; doi:10.2196/77233)
Supplement: Multimedia Appendix 4 [file jmir-v27-e77233-s004.docx]

**Supplementary file 4**

***Table 7:*** **Grade certainty table for each outcome of interest.**

| **Outcome** | **Study design** | **Risk of bias** | **Inconsistency** | **Indirectness** | **Imprecision** | **Publication bias** | **Upgrading factors**  **Total*** | **Overall certainty** |
| --- | --- | --- | --- | --- | --- | --- | --- | --- |
| BPSD | High | -1 | 0 | 0 | -1 | 0 | 0 | Low |
| BPSD-related caregiver burden | Moderate | -1 | 0 | 0 | 0 | 0 | 0 | Low |

**Legend:**

- Very large effect: +2 points
- Large effect: +1 point
- No concerns/no significant effect: 0 points
- Serious concerns: -1 points
- Very serious concerns: -2 points

*These upgrading factors are:

- Large magnitude of effect
- Dose response gradient
- Plausible residual confounding

**Footnote:**

1. **BPSD outcome reasoning:**

**Study design:** There were 8 RCTs, 2nRCTs, and 2 pre-post studies which contributed to the overall result of the analysis. The authors considered that the starting certainty rating for the body of evidence, based on study design, was high.

**Risk of bias:** Most studies were classified as fair quality, with 3 being classified as high quality, and 1 as low quality. Some studies, as observed in ***supplementary file 4***, lacked blinding, allocation concealment, or they did not report on other important factors to reduce bias. Importantly, the high-quality studies [28,31,38] did not provide conclusive evidence regarding the direction of intervention effects on BPSD outcomes. The certainty of evidence was therefore downgraded by one level.

**Inconsistency:** Heterogeneity in study findings was largely attributable to methodological differences, particularly in intervention design and follow-up timing. Variability in outcome measurement scales further explained differences in effect magnitude.

**Indirectness:** Despite the use of different measurements tools and the inclusion of studies only accessing specific symptoms, they were all outcomes of interest in this systematic review.

**Imprecision:** Although most studies suggested a positive trend and the overall sample size was adequate, higher-quality studies yielded inconclusive results. Furthermore, while some studies found statistically significant effects, others did not. The authors therefore downgraded for imprecision.

**Publication bias:** Although some individual studies had a small sample size, and the direction of results was consistently positive, grey literature search(ClinicalTrials.gov, and International Clinical Trials Registry Platform) did not reveal unpublished studies likely to alter the conclusions. Therefore, no downgrade was applied by the authors.

**Upgrading factors:**  The certainty of the evidence was not upgraded as there was no evidence of a very large effect, dose–response relationship, or residual confounding.

1. **BPSD-related caregiver burden outcome reasoning:**

**Study’ design:** There were 2 RCTs one of high-quality, one of fair-quality, 1 quasi-RCT of fair quality, and 2 pre-post intervention studies of fair quality, therefore, the GRADE certainty score would start with moderate.

**Risk of bias:** 4 out of the 5 studies were classified as fair quality according to the National Heart, Lung, and Blood Institute (NHLBI) tool from the National institute of health (NIH). Only one study was classified as high quality. Additionally, the pre–post studies did not report potential confounding factors between groups and did not employ multiple pre- and post-intervention measurements. This resulted in downgrading the certainty of evidence by one level.

**Inconsistency:** Differences in study effects could largely be explained by methodological heterogeneity, particularly in the intervention, and follow-up timings.

**Indirectness:** The included studies measured BPSD-related caregiver distress rather than overall caregiver burden. While not identical, the authors agreed this was the closest available measure. Equally, all studies used the same measurement scale (NPI-Q/NPI-Q-D).

**Imprecision:** The authors did not downgrade for imprecision as the total number of patients in the studies was adequate (234 participants for this specific measurement), and all studies demonstrated a positive effect of the interventions on BPSD-related caregiver burden.

**Publication bias:** Although some individual studies had a small sample size, and the direction of results was consistently positive, grey literature search(ClinicalTrials.gov, and International Clinical Trials Registry Platform) did not reveal unpublished studies likely to alter the conclusions. Therefore, no downgrade was applied.

**Upgrading factors:** The certainty of the evidence was not upgraded as there was no evidence of a very large effect, dose–response relationship, or residual confounding.
